# Protein phosphatases 1 and 2A and their naturally occurring inhibitors: current topics in smooth muscle physiology and chemical biology

**DOI:** 10.1007/s12576-017-0556-6

**Published:** 2017-07-05

**Authors:** Akira Takai, Masumi Eto, Katsuya Hirano, Kosuke Takeya, Toshiyuki Wakimoto, Masaru Watanabe

**Affiliations:** 1 Department of Physiology, Asahikawa Medical University, Midorigaoka-Higashi 2-1-1-1, Asahikwa, 078-8510 Japan; 2 Department of Molecular Physiology and Biophysics and Sidney Kimmel Medical College, Thomas Jefferson University, Philadelphia, PA 19107 USA; 3 Department of Cardiovascular Physiology, Faculty of Medicine, Kagawa University, 1750-1 Ikenobe, Miki-cho, Kita-gun, Kagawa 761-0793 Japan; 4 Faculty of Pharmaceutical Sciences, Hokkaido University, Kita 12, Nishi 6, Kita-ku, Sapporo, 060-0812 Japan; 5 Department of Frontier Health Sciences, Graduate School of Human Health Sciences, Tokyo Metropolitan University, Higashi-Ogu, Arakawa-ku 7-2-10, Tokyo, 116-8551 Japan

**Keywords:** Protein phosphorylation, Protein phosphatase 1, Protein phosphatase 2A, Smooth muscle, Phosphatase inhibitors, Signal transduction

## Abstract

Protein phosphatases 1 and 2A (PP1 and PP2A) are the most ubiquitous and abundant serine/threonine phosphatases in eukaryotic cells. They play fundamental roles in the regulation of various cellular functions. This review focuses on recent advances in the functional studies of these enzymes in the field of smooth muscle physiology. Many naturally occurring protein phosphatase inhibitors with different relative PP1/PP2A affinities have been discovered and are widely used as powerful research tools. Current topics in the chemical biology of PP1/PP2A inhibitors are introduced and discussed, highlighting the identification of the gene cluster responsible for the biosynthesis of calyculin A in a symbiont microorganism of a marine sponge.

## Introduction

Protein phosphatases 1 (PP1) and 2A (PP2A) are two of the principal catalytic subunits that dephosphorylate proteins on serine and threonine residues in the cytosol of eukaryotic cells. They are encoded by genes belonging to the PPP family and are abundantly expressed in all cell types. These enzymes achieve the substrate specificity and activity regulation required for their multiple functions through combinatorial interactions with many regulatory subunits [1].

Smooth muscle myosin light chain phosphatase (MLCP) is one of the PP1 holoenzymes whose molecular composition and functions are most intensively studied. The first section of this review assesses the current state of knowledge and issues related to the regulation of smooth muscle contraction by targeting and modulation of the MLCP activity. The second section to follow is related to the circadian oscillation of the MLCP activity in vascular smooth muscle. It summarizes the recent findings that elucidated how the oscillation is generated by the local intrinsic clock gene and discusses the physiological relevance of the circadian rhythmicity.

The third section concerns roles for PP2A in smooth muscles, about which little is known to date. During the last 3 decades since the debut of okadaic acid as an inhibitor of PP1 and PP2A, inhibitory effects on these enzymes have been demonstrated for other various naturally occurring substances with widely different structural features. Some of these, as well as okadaic acid itself, show exceedingly higher affinity to PP2A than to PP1. Possible involvement of PP2A in the regulation or maintenance of smooth muscle contraction will be considered on the basis of the recent findings obtained by experiments using such highly specific PP2A inhibitors.

The fourth and last section introduces current topics in the chemical biology of PP1/PP2A inhibitors, highlighting the identification of the gene cluster responsible for biosynthesis of calyculin A in a symbiont microorganism of the marine sponge *Discodermia calyx* from which the toxin was isolated. A detailed study has shown that the end product of the biosynthesis system is actually not calyculin A itself but a pyrophosphate protoxin (phosphocalyculin A) with a very weak inhibitory activity on PP2A, which is converted to calyculin A through dephosphorylation by phosphatase activity liberated upon tissue disruption of the sponge. Functional implications of this wound-activated bioconversion process will be discussed from the view point of chemical defense of the eukaryotic host organism by the symbiont-derived PP1/PP2A inhibitor.

Each section of this review is based on the four presentations given under the same titles in a symposium that we organized when the 93rd annual meeting of the Physiological Society of Japan was held in Sapporo from 22 to 24 March 2016 [2–5].

## An emerging paradigm shift for myosin phosphatase signaling in smooth muscles

**Fig. 1:**
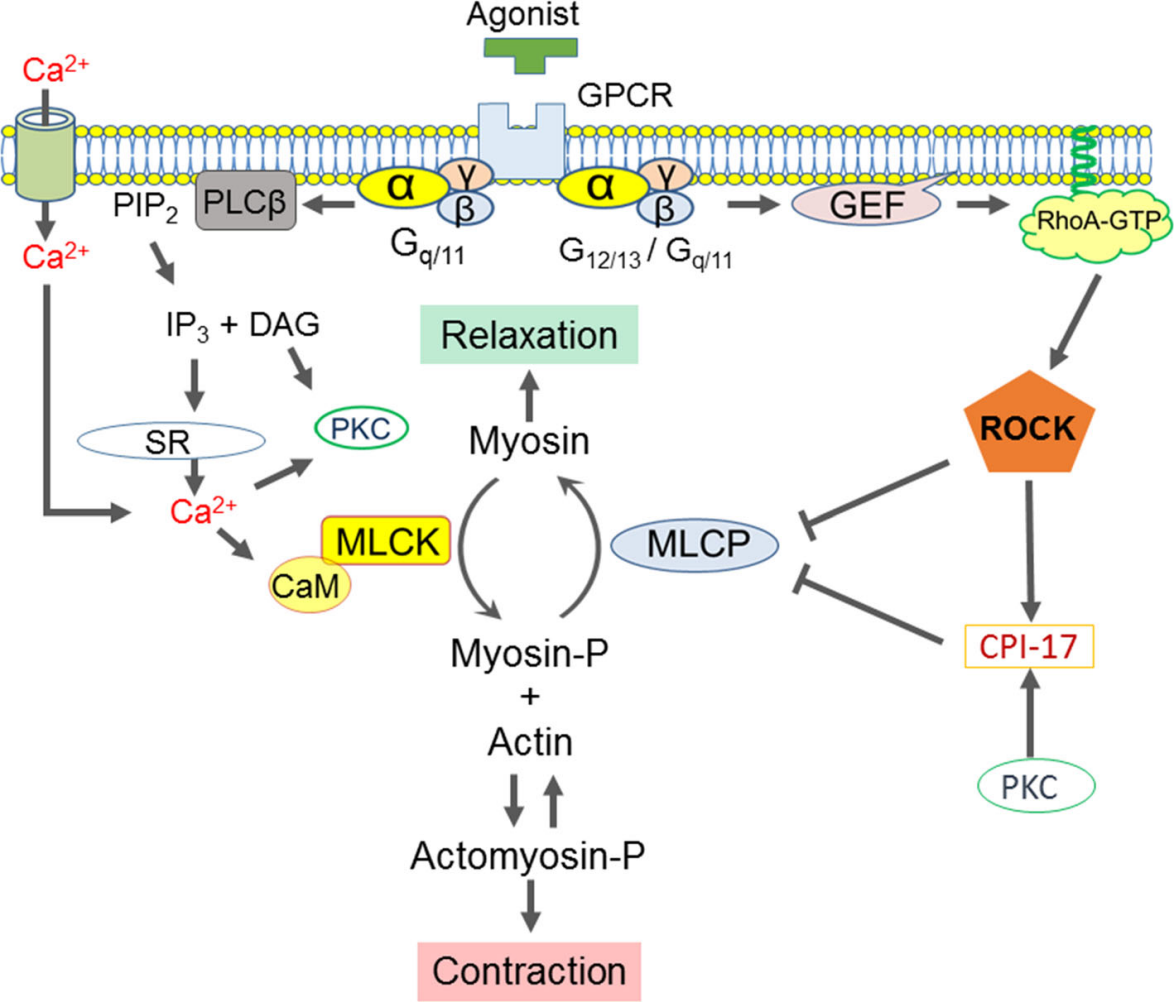
Current scheme for the regulation of smooth muscle contraction. *GPCR* G-protein coupled receptor; *PLC*β phospholipase Cβ; *PIP*_*2*_ phosphatidylinositol 4,5-bisphosphate; *IP*_*3*_ inositol 1,4,5-tripsphosphate; *DAG* diacylglycerol; *PKC* protein phosphatase C; *SR* sarcoplasmic reticulum; *CaM* calmodulin; *MLCK* myosin light chain kinase; *MLCP* myosin light chain phosphatase; *GEF* guanine nucleotide exchange factor; *ROCK* RhoA/rho-associated coiled-coil-containing protein kinase; *CPI*-17 PKC-potentiated inhibitory protein of 17 kDa. See the text for details

Reversible phosphorylation of the 20-kDa myosin regulatory light chain (MLC) governs cell motility, including smooth muscle force development, which is regulated through multiple pathways specific to each cell type. In smooth muscles, the Ca^2+^/CaM-dependent myosin light chain kinase (MLCK) plays a primary role in phosphorylating MLC (at Thr18 and Ser19 with a preference for the latter) in response to an elevation of the cytoplasmic free Ca^2+^ concentration, [Ca^2+^]_*i*_ [6, 7] (Fig. 1). Regulation of [Ca^2+^]_*i*_ has thus been a research focus for understanding the excitation-contraction coupling in each smooth muscle type.

**Fig. 2:**
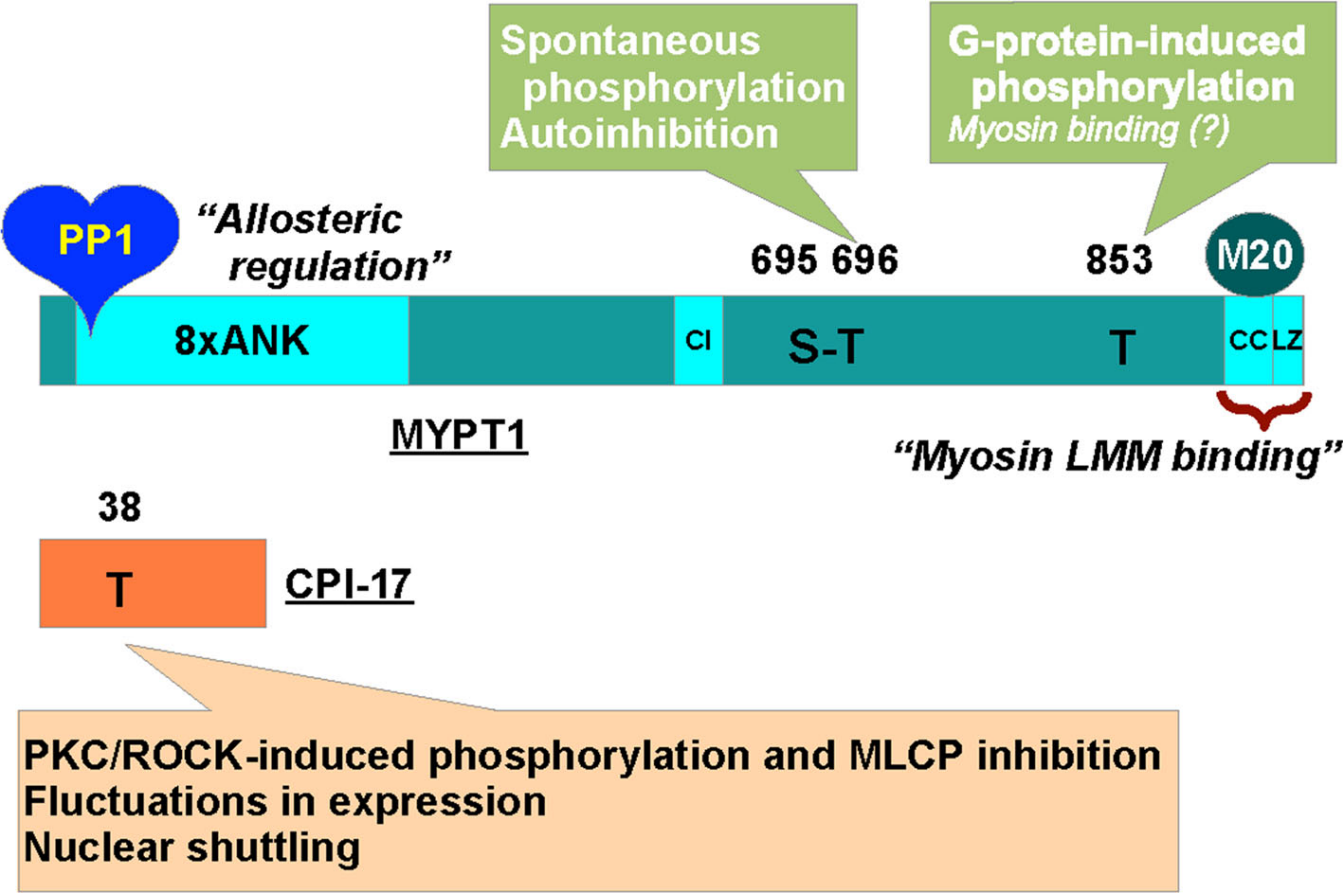
The structure of the myosin light chain phosphatase (MLCP) and that of the endogenous regulatory protein CPI-17. *PP1* a type-1 phosphatase catalytic subunit (PP1; β/δ isoform), *MYPT1* myosin phosphatase target subunit 1, *8× ANK* 8-repeat ankyrin motif, *CI* central insert domain, *LZ* Leu-zipper domain, *CC* coiled-coil domain, *M20* the 20-MDa accessory subunit, *LMM* light meromyosin. See also the legend to Fig. 1

Growing lines of evidence suggest that modulation of MLCP activity is another dominant mechanism that determines the velocity and extent of smooth muscle contraction in response to stimuli (Fig. 1) [8–10]. MLCP is a heterotrimeric holoenzyme, consisting of the catalytic subunit PP1 (β/δ isoform) and the regulatory complex myosin phosphatase targeting subunit 1 (MYPT1) associated with an accessory subunit M20 (Fig. 2) [8–10]. Many PP1-binding proteins contain a KVXF motif, which interacts with a hydrophobic pocket of PP1 and therefore plays an important role in PP1 binding [11]. This motif is found in residues 35–38 of human MYPT1 and serves as a primary binding site for PP1 [8, 9]. PP1 docks at the N-terminal ankyrin-repeat domain (8× ANK) of MYPT1, which confers substrate specificity through allosteric regulation (Fig. 2) [8, 12]. The MYPT1 C-terminal coiled-coil (CC) and Leu-zipper (LZ) domains bind to the light meromyosin (LMM) region of myosin, tethering PP1 to actomyosin filaments [13, 14]. The assembly of PP1 with MYPT1 accelates myosin dephosphorylation in smooth muscle skinned fibers [15]. Alternative splicing of the PPP1R12A gene transcript results in MYPT1 variants with or without the central insert (CI) and LZ domain, which contribute to defining smooth muscle responsiveness to elevations in cytoplasmic GTP and cGMP, respectively (Fig. 2) [16, 17]. The 20-kDa accessory subunit (M20) docks at the MYPT1 C-terminal helical domain (Fig. 2) [18] and is co-purified with MYPT1 and PP1 from smooth muscle homogenates [19], although the M20 binding has little effect on the phosphatase activity [14].

There is a mass of evidence to show that post-translational modification of MYPT1, such as phosphorylation [9, 10, 20] and others [21–23], regulates cellular MLCP activity in response to physiological and experimental stimuli. Two phosphorylation sites, Thr696 and Thr853, of MYPT1 (numbering based on human PPP1R12A gene) have drawn the most attention in the smooth muscle physiology. The first report [24] showed that stimulation of skinned portal vein strips with ATPγS induced an elevation of MYPT1 phosphorylation in parallel with a decrease in MLCP activity, although the phosphorylation site involved in the inactivation was then unidentified. It was soon shown that the activity of MLCP isolated from tissues was suppressed when MYPT1 Thr696 was phosphorylated by a kinase associated with MLCP [25]. Later, the MLCP-associated kinase was identified as a variant of zipper interacting protein kinase (ZIPK) [26]. Independently, RhoA-associated coiled-coil-containing protein kinase (ROCK) was reported to directly phosphorylate recombinant MYPT1 at both Thr696 and Thr853 [13, 27]. These data were the foundations for establishing the current paradigm of the RhoA/ROCK-mediated MLCP inactivation—a mechanism transducing stimulation of G-protein coupled receptors (GPCRs) into phosphorylation of MLC and contraction of smooth muscle [20, 28]. Multiple Rho guanine nucleotide exchange factors, such as PDZ-RhoGEF and LARG, are suggested to mediate between GPCR and RhoA in smooth muscles [29]. It should also be noted that this RhoA/ROCK-MLCP signaling axis has been applied to interpreting other cellular events, becoming a well-accepted pathway in the cell signaling study field [30]. For example, RhoA/ROCK-mediated modulation of MLCP activity has been shown to play indispensable roles in the embryonal development of model animals such as worm [31], fly [32] and mouse [33].

Nonetheless, emerging evidence suggests exceptions to the RhoA/ROCK-mediated MLCP inactivation paradigm [34]. Multiple reports showed that MYPT1 Thr696 was spontaneously phosphorylated and barely elevated in mature smooth muscles upon stimulation [35–37], suggesting that Thr696 phosphorylation is not the target of RhoA/ROCK nor responsible for the GTPγS-induced MLCP inactivation in smooth muscles. The persistent phosphorylation of MYPT1 at Thr696 is attributed to insufficient auto-dephosphorylation [14]. Because phosphorylation of MYPT1 at Thr696 reduced MLCP activity to 20–30% through autoinhibition [14, 38, 39], a fraction of cellular MLCP must be persistently inactivated. On the other hand, MYPT1 Thr853 phosphorylation was low under resting conditions and was elevated in response to G-protein activation in parallel with contraction [35–37]. There is no doubt that MYPT1 Thr853 phosphorylation indicates cellular ROCK activity. In recombinant MLCP preparations, MYPT1 Thr853 phosphorylation interfered with its binding to reconstituted myosin filaments and myosin in cell cultures [13, 14], but it had little effect on the phosphatase activity [14, 38]. Also, recent reports using MYPT1 transgenic mice showed that Thr696 substitution desensitized the contractility of the bladder and ileum probably because of MLCP activation, whereas in contrast Ala-substitution of MYPT1 at Thr853 caused negligible changes in the morphology of bladder and ileum walls and had no effect on physiological functions of the smooth muscle strips from these tissues [40, 41]. The subtle change in myosin binding upon Thr853 phosphorylation may have only a limited impact on cellular MLCP activity in mature smooth muscle crowded with actomyosin filaments. Thus, MYPT1 phosphorylation at Thr696 or Thr853 is unlikely to mediate the GTPγS-induced MLCP inactivation at least in bladder and ileum smooth muscles. Roles of Thr853 phosphorylation in other smooth muscle tissues, such as artery and airway, and non-smooth muscle cells have yet to be determined.

It should also be noted that effects of smooth muscle-specific deletion of the MYPT1 gene on excitation–contraction coupling depend on smooth muscle types and experimental conditions. For example, conditional genetic deletion of MYPT1 augmented vasoconstriction and elevated blood pressure and suppressed the dephosphorylation of MLC and the relaxation of ileal smooth muscle [41, 42]. On the other hand, MYPT1 deletion showed less impact in carbachol-induced contraction of bladder smooth muscle [43]. Interestingly, MLC dephosphorylation and smooth muscle relaxation occurred in MYPT1-null smooth muscle strips, suggesting that there are other phosphatases that can compensate for the MLCP deficiency. Furthermore, MYPT1 deletion attenuated ROCK-induced contraction in the ileum and bladder and to a lesser extent in artery [41–43].

These recent observations support but also show exceptions to the status quo concept—MYPT phosphorylation regulates smooth muscle regulation. However, before drawing any conclusion, it should be noted that there is a caveat against this argument, which involves the potential misreading of MYPT1 phosphorylation data due to the sensitivity of the phospho-specific antibodies. MacDonald’s group showed that MYPT1 phosphorylation at the adjacent Ser residues, Ser695 and Ser852, fluctuated depending on conditions [44]. Although Ser695 phosphorylation was not inhibitory [39], it could interfere with immunoblotting signals with antibodies for phospho-MYPT1 Thr696 [44]. Therefore, the current data of MYPT1 Thr696 phosphorylation as the foundation of our knowledge are likely to be influenced by the phosphorylation status of Ser695. The other possibility is that the spontaneous autoinhibition by phospho-Thr696 is regulated through other factors, such as Par4 and telokin [45, 46], without changing the phosphorylation status. Also, phosphorylation-induced translocation of MYPT1 may play a role [47]. To gain a clearer view of GPCR-induced smooth muscle regulation, MYPT1 Thr696 phosphorylation must be carefully re-evaluated using better means of quantification.

Another critical player in MLCP regulation is protein kinase C (PKC)-potentiated inhibitory protein of 17 kDa (CPI-17), a product of the PPP1R14A gene that functions as an endogenous MLCP regulator (Fig. 2) [48–50]. There are three layers of regulation for the CPI-17 signaling that determine the responsiveness of smooth muscle. First, CPI-17 potently inhibits MLCP when it is phosphorylated at Thr38 by ROCK and PKC (Fig. 1) [48, 49, 51]. Upon GPCR stimulation, PKC is quickly and transiently activated in parallel with Ca^2+^ elevation and withdrawal, followed by a slow and steady activation of ROCK (Fig. 1) [52]. This biphasic activation of two kinases determines the velocity and extent of CPI-17 phosphorylation and smooth muscle force development. Second, CPI-17 expression levels are linked to the characteristics of smooth muscles [53, 54]. For example, CPI-17 expression is higher in smooth muscles classified into the tonic type, such as artery, bronchus and sphincters, compared with phasic muscles, such as bladder and vas deferens [53]. Also, the lack of the CPI-17 gene in farm chicken results in lesser responsiveness of the smooth muscle compared with tissues from other species expressing the protein [55]. Notably, the CPI-17 deficiency in chicken smooth muscle seems to be compensated with an elevated Ca^2+^ sensitivity. CPI-17 expression fluctuates in response to pathophysiological stimuli, altering the responsiveness of the smooth muscle [56, 57]. For example, CPI-17 down- and upregulation in inflamed smooth muscle have been linked to hypo- and hyper-responsiveness in inflammatory bowel disease [58] and asthma [59], respectively. Third, upon pathological de-differentiation of smooth muscle cells, such as in neointimal formation observed in arteries following injury, CPI-17 translocates from the cytoplasm to the nuclei, regulating histone phosphorylation and cell growth independently from MLCP signaling [60]. These lines of evidence strongly suggest that three elements in the signaling, namely, phosphorylation, expression and localization of CPI-17, synergistically contribute to defining smooth muscle characteristics, supporting the concept that CPI-17 is a functional marker of smooth muscle [61].

MLCP is a multifunctional regulator of various cells, yet our knowledge is incomplete [30]. Cell-permeable antagonists for serine/threonine phosphatases, such as okadaic acid and calyculin A (see “Effects of PP2A inhibitors on smooth muscle contractio” and “Recent topics in sponge-derived protein phosphatase inhibitors” sections), are powerful tools for studying their physiological and pathophysiological roles. In the smooth muscle physiology field, these inhibitor compounds have contributed to establishing the myosin phosphorylation theory of excitation-contraction coupling [62, 63], to identifying and characterizing abnormal phosphorylation under pathological conditions [64] and to the discovery of new regulatory circuits in smooth muscle regulation [65, 66] (see “Effects of PP2A inhibitors on smooth muscle contraction” section). Nonetheless, the limitation of these antagonists lies in their lack of ability to distinguish MLCP among over 300 cellular serine/threonine phosphatase holoenzymes, each of which regulates a specific subset of protein phosphorylation. One possible solution is to improve specificity for MLCP by chemical engineering of inhibitor compounds. An alternative approach is to use CPI-17, which can distinguish MLCP from other cellular phosphatases [67]. To make use of CPI-17 as a new research tool for this purpose, it needs to be redesigned for stabilizing the inhibitory potency induced by Thr38 phosphorylation. Unfortunately, neither Asp- nor Glu-substitution of CPI-17 at Thr38 is capable of functionally mimicking the phosphorylation at all [68]. A better understanding of the molecular basis of CPI-17 functions is needed for establishing a new probe for cellular MLCP.

## Vascular intrinsic circadian rhythm of MLCP activity

The physiological parameters of the cardiovascular system, such as blood pressure and heart rate, as well as the occurrence of cardiovascular events, such as ischemic heart attack and myocardial infraction, show diurnal rhythms [69, 70]. The circadian rhythms of the sympathetic nerve activity, endothelial function, platelet function, coagulation/fibrinolysis activity and vascular contractility appear to contribute to the physiological and pathophysiological circadian events of the cardiovascular system. The vascular cells express a molecular mechanism that acts as a biological clock, which is similar to that found in the neuronal cells of the central clock located in suprachiasmatic nuclei [71]. The circadian rhythm of vascular functions is therefore considered to be determined by interplay between the central and vascular clocks. Clock gene products are transcription factors. Their mutual feedback loop network is essential for generating the circadian rhythm of their expression [72]. The rhythm of this core clock system is then translated to the rhythm of an oscillator molecule, which generates the circadian rhythm of cellular functions. However, the underlying molecular mechanism by which the intrinsic clock system generates the circadian rhythm of vascular contractility remains largely unknown.

**Fig. 3:**
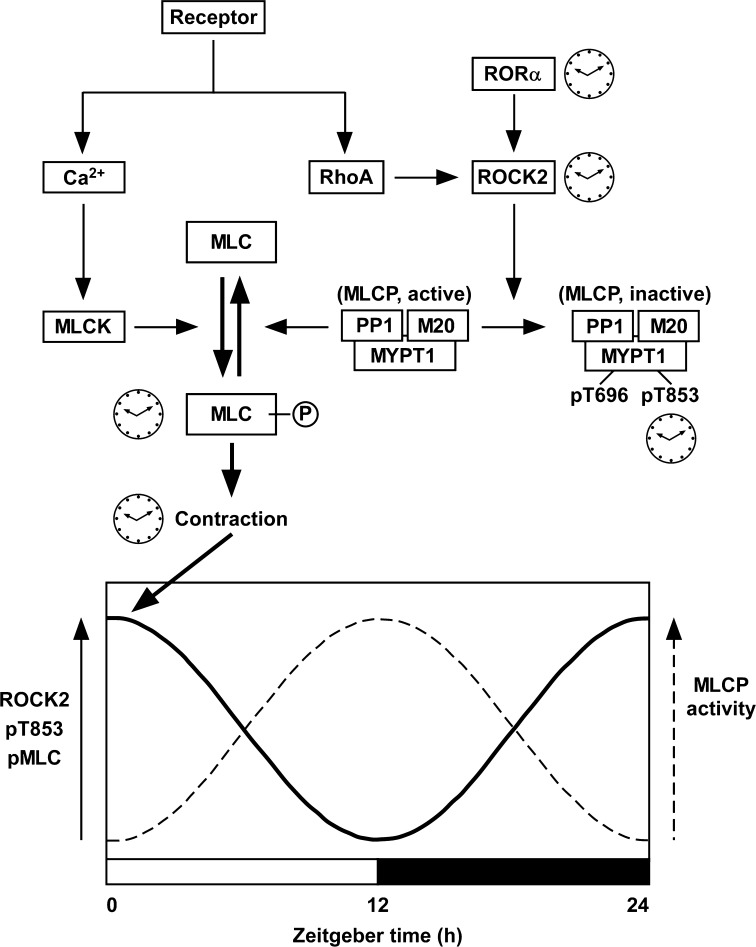
Schematic presentation of the mechanism of the vascular intrinsic clock that generates the circadian rhythm of the activity of myosin light chain phosphatase (MLCP). The circadian oscillation of the expression of the clock gene RORα is translated to the rhythmic changes in the expression and activity of ROCK2, which in turn causes the oscillatory changes in the phosphorylation of MYPT1, a regulatory subunit of MLCP, at Thr853 (pT853) and the phosphorylation of MCL (MLC-P) induced by receptor agonist, thereby generating diurnal changes in the vascular contractility. The processes, which exhibited a circadian rhythm in [20], are indicated by clock images. MLCP is composed of three subunits: a catalytic subunit PP1, MYPT1 and another regulatory subunit M20. The activity of MLCP is negatively regulated by the phosphorylation of MYPT1 at either Thr696 (pT696) or T853 (pT853). Therefore, when the expression of ROCK2 and the phosphorylation of MYPT1 are high (*solid line curve*), the activity of MLCP is considered to be reciprocally low (*dashed line curve*). *MLC* 20-kDa myosin light chain, *ROR*α retinoic acid receptor-related orphan receptor α (see also Figs. 1, 2)

Recently, Saito et al. [73] identified ROCK type 2 (ROCK2) as an oscillator molecule generating the circadian rhythm of vascular contractility (Fig. 3). Further, they showed that ROCK2 was directly controlled by the clock gene retinoic acid receptor-related orphan receptor α (RORα) [73]. In cultured vascular smooth muscle cells, agonist-induced phosphorylation of MLC, but not the resting level of phosphorylation, exhibited circadian oscillation after synchronization of the biological clock by dexamethasone pulse treatment [73]. The rhythmic change in the MLC phosphorylation was abolished by inhibiting ROCK2 activity via a pharmacological inhibitor or genetic knockdown. Indeed, the phosphorylation of MYPT1 at T853, which reflects the activity of ROCK2, as well as the expression of ROCK2 protein oscillated in phase with the level of MLC phosphorylation [73]. However, the activity of RhoA, an upstream regulator of ROCK2, or the expression of other kinases involved in MLC phosphorylation, such as MLCK, PKC and ZIP kinase, did not show any circadian oscillation [73]. ROCK2 therefore appeared to be a direct target of the vascular clock. The promoter region of the ROCK2 gene was found to contain a tandem repeat of a ROR-response element. This arrangement of the promoter region is conserved among several mammalian species. A luciferase-based promoter assay showed that RORα and RORγ activated the ROCK2 gene promoter. However, the expression of RORα, but not of RORγ, exhibited circadian oscillation in phase with the expression of ROCK2 mRNA. Furthermore, gene silencing of RORα abolished the rhythmic change in ROCK2 expression. These observations with cultured smooth muscle cells collectively suggest that RORα generates the circadian rhythm of the expression and activity of ROCK2, which causes the oscillation of MLC phosphorylation. ROCK2 regulates MLC phosphorylation by either directly phosphorylating MLC in a Ca^2+^-independent manner or indirectly inactivating MLCP activity via MYPT1 phosphorylation. Since the resting level of MLC phosphorylation exhibited no circadian rhythm and ROCK2 has a higher substrate preference for MYPT1 to MLC, the oscillation of the MLCP activity plays a major role in the ROCK2-mediated generation of the circadian rhythm of MLC phosphorylation.

This notion obtained from experiments with cultured smooth muscle cells was corroborated by experiments with RORα-deficient natural mutant *staggerer* mice [74]. In the aorta of wild-type mice, the expression of ROCK2 exhibited circadian oscillation with a zenith at zeitgeber time (ZT) 0/24 and a nadir at ZT12 (Fig. 3). The circadian oscillation of ROCK2 expression was absent in *staggerer* mice. In the membrane-permeabilized preparations of the aorta of wild-type mice, the Ca^2+^-induced contraction at ZT0/24 was similar to that seen at ZT12. However, the Ca^2+^-sensitizing effects induced by the non-hydrolyzable GTP analog GTPγS and the thromboxane A_2_ receptor agonist U46619 at ZT0/24 were greater than those observed at ZT12. The MLC phosphorylation induced by U46619 at ZT0/24 was also greater than that seen at ZT12. All of these diurnal changes were absent in *staggerer* mice. Although the level of phosphorylation of MYPT1 at T853 was not directly addressed in mouse aorta, the ROCK2-mediated oscillation of MLCP activity may play a critical role in generating the circadian rhythm of MLC phosphorylation, myofilament Ca^2+^ sensitivity and vascular contractility (Fig. 3).

These findings from the experiments with cultured smooth muscle cells and *staggerer* mice consistently suggest that RORα plays an essential role in the vascular intrinsic clock mechanism that generates the circadian rhythm of vascular contractility. According to a proposed scheme (Fig. 3), RORα directly regulates the circadian rhythm of the expression and activity of ROCK2, which then causes circadian oscillation of MYPT1 phosphorylation at T853 and MLC phosphorylation. Although the role of T853 phosphorylation in the regulation of MLCP activity remains controversial (see “An emerging paradigm shift for myosin phosphatase signaling in smooth muscles” section above), the circadian oscillation of the MLCP activity is suggested to play a key role in the ROCK2-mediated circadian oscillation of MLC phosphorylation (Fig. 3).

The diurnal change in the vascular contractility was consistently observed in the earlier study by Su et al. [75]. The contractile responses to phenylephrine, high K^+^ and angiotensin II in the isolated mice aortas as well as the in vivo pressor responses to phenylephrine and angiotensin II were high in the light phase and low in the dark phase under physiological conditions. However, the underlying molecular mechanism remains unclear. The mRNA expression of ROCK1, ROCK2, CPI-17 and RhoA exhibited a diurnal change, with a zenith at ZT17-23. This circadian rhythm is out of phase with that seen for the contractile response, which reached a nadir at ZT17. The observations by Su et al. [75] were also in apparent contrast to those in the study by Saito et al. [73], which showed no diurnal change in the protein expression of ROCK1 or the RhoA activity. The reason for this discrepancy is also unknown.

The physiological relevance of the identified vascular intrinsic clock still remains to be investigated. Since mice are nocturnal and humans are diurnal, the extrapolation of the observations in mice to human physiology requires caution. For example, the ROCK2-mediated circadian rhythm of vascular contractility with a zenith in the light period in mice may be translated to the circadian rhythm with a zenith in the dark period in humans. In this case, the vascular intrinsic circadian rhythm does not appear to be directly related to the physiological circadian rhythm of blood pressure or the diurnal oscillation of cardiovascular events in humans, which attain their zeniths in the early morning. However, a recent human study investigated the endogenous rhythm of blood pressure by subjecting participants to three different protocols of desynchrony to remove confounding behavioral and environmental factors [76]. The results of three different protocols consistently indicated the existence of endogenous circadian rhythms of blood pressure, which surprisingly reached a zenith at the circadian phase corresponding to 9 p.m. The vascular intrinsic clock as the one found in mice may underlie the mechanism for the endogenous circadian rhythm of blood pressure in humans. On the other hand, the circadian rhythm of blood pressure under pathological conditions and the diurnal oscillation of cardiovascular events may not be directly caused by the augmentation of the physiological rhythm. Instead, the dysregulation of the physiological rhythm may underlie the loss of the diurnal blood pressure rhythm and increased risk of cardiovascular events under pathological situations. In fact, Su et al. [75] demonstrated that the circadian rhythm of vascular contractility and the expression of ROCK1, ROCK2 and CPI-17 seen in the control mice was abolished in diabetic db/db mice. Future studies are expected to clarify the physiological and pathophysiological relevance of the intrinsic circadian rhythm of vascular contractility.

## Effects of PP2A inhibitors on smooth muscle contraction

**Fig. 4:**
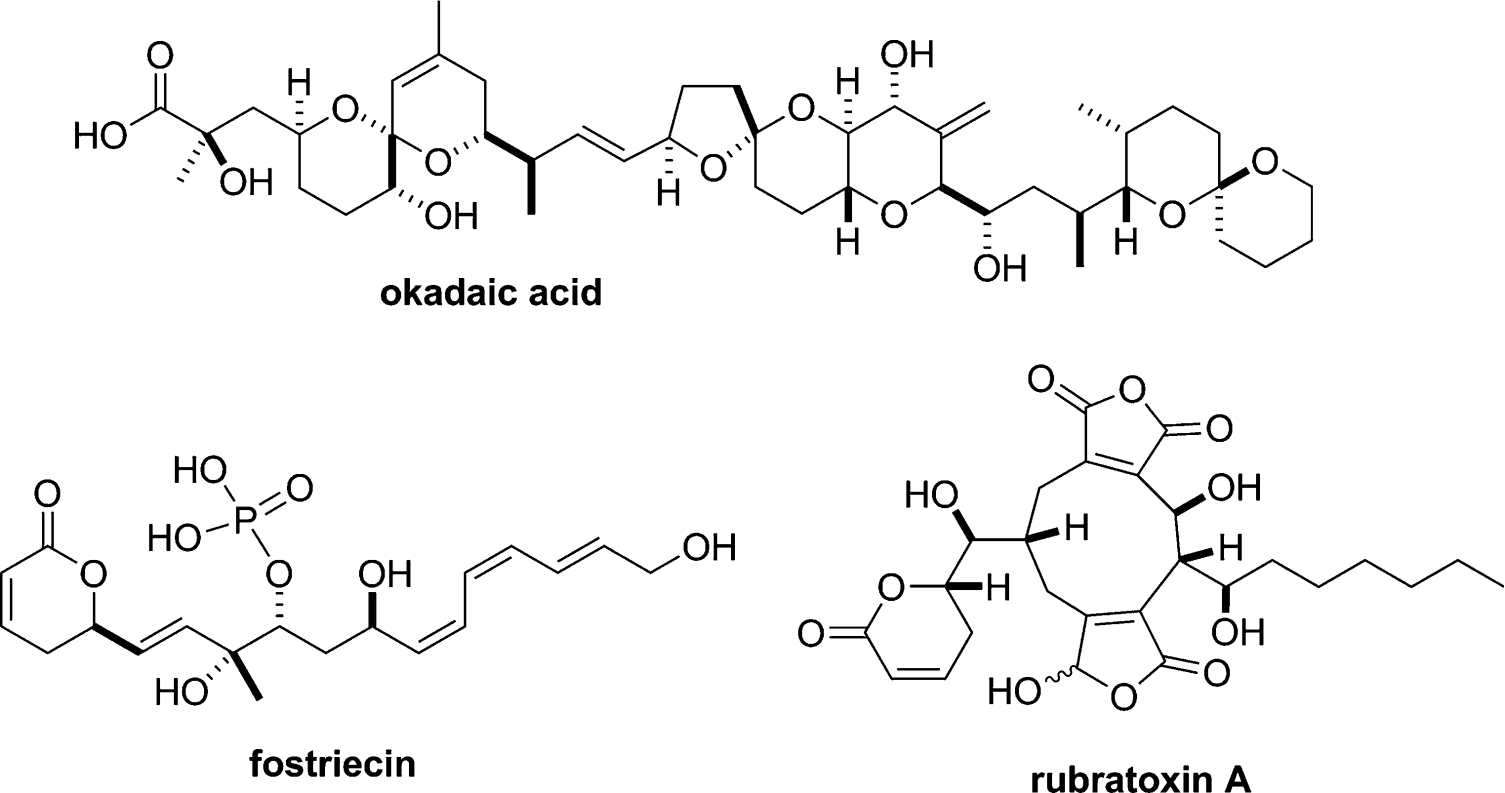
Chemical structures of PP2A inhibitors—okadaic acid, fostriecin and rubratoxin A

During the last 3 decades since okadaic acid (Fig. 4) was shown to be an inhibitor of PP1 and PP2A [62, 77–79], inhibitory effects on these enzymes have been reported for various other naturally occurring substances with different relative affinities to PP1 and PP2A [80–93] (Table 1; see “Recent topics in sponge-derived protein phosphatase inhibitors” section below for the natural sources of these substances). Since these inhibitors increase intracellular phosphorylation in various types of cells in culture (mostly at 37 °C) in the manner expected of PP1/PP2A inhibitors [89, 93–100], they are generally believed to be membrane permeable (except for microcystin-LR and its relatives, which are not readily taken up by most cell types [101]), although it should be noted that the membrane permeability of the inhibitors may be drastically affected by such factors as the temperature or tissue-specific lipid composition of the cell membrane [102]. They are now widely used as valuable tools in physiological as well as biochemical research fields.

**Table 1:** Inhibition of PP1 and PP2A by some naturally occurring inhibitors

Inhibitors	*K*_i_ (nM)		References
	PP1	PP2A	
Okadaic acid	150	0.032	80–82
Calyculin A	1	0.13	82, (83, 84)
Tautomycin	0.5	30	82, (85)
Microcystin-LR	0.22	0.008	82, (86, 87)
Nodularin^c^	0.11^a^	0.022^a^	(88)
Motuporin^d^	<1^b^	n.d.	(89)
Cantharidin	470^b^	40^b^	(90, 91)
Fostriecin	130 μM^b^	3^b^	(92)
Rubratoxin A	>200 μM^b^	30^b^	(93)

The dissociation constants (*K*_i_) for the interaction of PP1 and PP2A with the naturally occurring inhibitors referred to in this review are listed. The values are in nM, unless otherwise stated. The difference between *K*_i_ and ID_50_ is usually negligible for inhibitors with relatively low affinity (say, *K*_i_ > 100 nM)

*n.d.* not determined

^a^Unpublished *K*_i_ data of A. Takai

^b^ID_50_ instead of unavailable *K*_i_ data. The references in parentheses present ID_50_, which is generally dependent on the concentration of phosphatase in the assay (see Takai and Mieskes [80])

^c,d^Also called nodularin-R and nodularin-V, respectively

It was the force-enhancing effect of okadaic acid on Triton-X100-skinned smooth muscle fibers that provided an initial clue to the discovery of its inhibitory action to PP1 and PP2A [62, 78, 79, 103]. However it was soon shown that, when applied at 30–37 °C to preparations with intact cell membrane, relatively low concentrations (<3 μM) of okadaic acid strongly inhibited rather than enhanced contractions induced by agonist stimulation or high K^+^ depolarization. Since the initial reports in 1989 [104, 105], the relaxant effect of okadaic acid has been demonstrated (at 30–37 °C; for the temperature dependence of this effect, see below) in a variety of smooth muscles including rabbit aorta [104]; dog basilar artery [105]; pig coronary artery [105]; bovine trachea [106] and iris [107]; and guinea pig vas deferens [108], taenia caeci [66, 109], ileum and pulmonary artery (A. Takai, unpublished observations). However, okadaic acid (0.1–3 μM) failed to inhibit the carbachol-induced contraction in bovine ciliary muscle [66]. To our knowledge this is hitherto the only reported case in which a smooth muscle with intact cell membrane escaped the relaxant effect of okadaic acid.

At higher concentrations (>10 μM), okadaic acid was constantly shown to produce or enhance contractions in intact smooth muscle preparations [107, 110] as well as in permeabilized preparations [62, 65, 78, 111, 112]. Okadaic acid (10 and 30 μM) showed a clear enhancing effect even on the CCh-induced contraction of bovine ciliary muscle [66], which was not inhibited by lower concentrations of the toxin (see above).

The contractile effects of okadaic acid can most simply be explained by inhibition of MLCP, which contains PP1 as the catalytic subunit (see “An emerging paradigm shift for myosin phosphatase signaling in smooth muscles” section above) [109]. On the other hand, there is increasing evidence to indicate that the relaxant effect of okadaic acid on most intact smooth muscles is produced by inhibition of PP2A.

The relaxant effect is highly dependent on temperature. In the intact smooth muscle bundle of the porcine coronary artery, for example, 3 μM okadaic acid, which abolished K^+^-induced contracture at 37 °C high (40 mM), produced no change in the tension when applied at 30 °C [105]. In rat uterine muscle permeabilized with α-toxin, however, it was shown that contraction induced at 26 °C by submaximal concentrations of Ca^2+^ was strongly suppressed by okadaic acid at 30–300 nM, whereas it was enhanced at 1–10 μM [65]. In the same permeabilized preparation, methyl okadaate and norokadanone, two okadaic acid derivatives without inhibitory action on PP1 and PP2A [113], caused neither suppression nor enhancement of the Ca^2+^-induced contraction [65]. In guinea pig portal vein, the relaxing effect was not observed in muscle fibers chemically skinned using Triton-X100 or β-escin, whereas it was clearly demonstrated even at 25 °C when α-toxin was used instead for skinning [114]. These observations are consistent with the notion that the relaxant effect of okadaic acid is not produced by some non-specific action of the toxin but by inhibition of a certain fraction of intracellular protein phosphatase activities that is lost by intensive permeabilization of the cell membrane with detergents such as β-escin or Triton-X100. Because the rate constants for physical permeation of various hydrophilic and lipophilic solutes through the lipid bilayer of the cell membrane are known to exhibit steep temperature dependence with *Q*_10_ values much larger than unity [115], it seems reasonable to interpret the ineffectiveness of okadaic acid toward intact (not permeabilized) preparations at lower temperature as being due at least in part to a reduction of the membrane permeability.

Okadaic acid has much higher affinity to PP2A (*K*_i_ = 32 pM) than to PP1 (*K*_i_ = 147 nM) [80, 82]. Also, it was shown that the fraction of the protein phosphatase activity showing a high sensitivity (*K*_i_ = 30–50 pM) to okadaic acid was lost in Triton-X100-skinned smooth muscles [116], the contractility of which was not inhibited by okadaic acid (see above). It is therefore natural to assume that the relaxant effect of okadaic acid on intact smooth muscles is produced by specific inhibition of PP2A. This assumption was recently supported by Ishida et al. [4, 66, 117], who examined the effects of other PP2A inhibitors on contractions of guinea pig taenia caeci and bovine ciliary muscle. For this purpose, they chose fostriecin [92] and rubratoxin A [93] because both of these protein phosphatase inhibitors also show exceedingly higher affinity to PP2A than to PP1 (Table 1). In guinea pig taenia caeci and bovine ciliary muscle, Ishida et al. [4, 66, 117] showed that fostriecin and rubratoxin A, as well as okadaic acid, dose-dependently relaxed the contractions induced by a Ca^2+^ ionophore, ionomycin. The three toxins also strongly inhibited the carbachol-induced contraction in guinea pig taenia caeci [66]. Since the three PP2A inhibitors have widely different structures (Fig. 4), they concluded that their relaxing effects were most likely to be produced by inhibition of PP2A rather than by some non-specific actions. This conclusion has an important implication that some fraction of PP2A is somehow involved in the regulation or maintenance of smooth muscle contraction.

Interestingly, however, okadaic acid failed to inhibit the carbachol-induced contraction in bovine ciliary muscle [66] (see above). In this smooth muscle, fostriecin also failed to relax the carbachol-induced contraction, which was inhibited by rubratoxin A as strongly as the one induced by ionomycin [66]. What makes the carbachol-induced contraction in bovine ciliary muscle resistant to the relaxant effect of okadaic acid and fostriecin is so far not known. Fostriecin and rubratoxin A, unlike okadaic acid, showed no contractile effect in guinea pig taenia caeci and bovine ciliary muscle even at higher concentrations [66]. This is expected because, in the concentration range (up to 30 μM) used in the experiments, fostriecin and rubratoxin A have only very weak or practically no inhibitory effect on PP1 [92, 93].

If we are to accept that inhibition of PP2A is the initial step for the toxins to produce their relaxant effects, then what are the subsequent steps to lead to suppression of the contractility? At present our knowledge about physiological functions of PP2A in smooth muscles is generally very limited and fragmental, and there is as yet no clear answer to this question. In addition to CPI-17 [118] (see “An emerging paradigm shift for myosin phosphatase signaling in smooth muscles” section), several smooth muscle proteins are suggested to be physiological substrates for PP2A, including calponin [119], caldesmon [120], the L-type Ca channel [121] and large-conductance Ca^2+^-activated potassium (BK) channel [122]. However, the proposed actions of PP2A-dephosphorylated forms of these proteins on smooth muscle contractility are all relaxant; inhibition of PP2A would cause elevation of their phosphorylation and result in smooth muscle contraction rather than relaxation.

In their experiments in canine basilar artery, Obara et al. [123, 124] showed that inhibitors of PKCα attenuated the inhibitory effect of okadaic acid on high K^+^ (80 mM)-induced contraction. They speculated that unmasking of the basal PKCα activity to induce triple phosphorylation of 20-kDa MLC was the cause of the inhibitory effect of okadaic acid [123, 124]. In guinea pig taenia caeci and bovine ciliary muscle, however, it was shown that the relaxant effect of okadaic acid on ionomycin-induced contraction was not affected by 1 μM of Gö6983 [66] and Gö6976 (K. Takeya, unpublished observation), PKC inhibitors that inhibit PKC isozymes including PKCα at nanomolar concentrations [125]. Examination of the effect of PKC inhibitors on the relaxations induced by the PP2A inhibitors in other smooth muscles would be interesting.

In most cases reported, the relaxant effect of okadaic acid was not accompanied by a marked reduction of the [Ca^2+^]_*i*_. A slight reduction of [Ca^2+^]_*i*_ was observed on application of okadaic acid in some types of smooth muscle [104, 105]. However, the [Ca^2+^]_*i*_ was reduced by only 10–20% even when the contraction was almost completely inhibited by okadaic acid [104, 105]. The relaxation produced by okadaic acid does not appear to be well correlated with a reduction of the MLC phosphorylation. In bovine trachea [106] and guinea pig taenia caeci [109], MLC remained highly phosphorylated when the contractility was lost by treatment with okadaic acid. These observations may suggest that the physiological role for the PP2A fraction involved in okadaic acid-induced relaxation is related to the regulation of rather downstream steps of smooth muscle contraction, such as cross-bridge cycling and cytoskeletal reorganization [126]. Further studies are expected to elucidate the detail of the function of PP2A in smooth muscles.

## Recent topics in sponge-derived protein phosphatase inhibitors

**Fig. 5:**
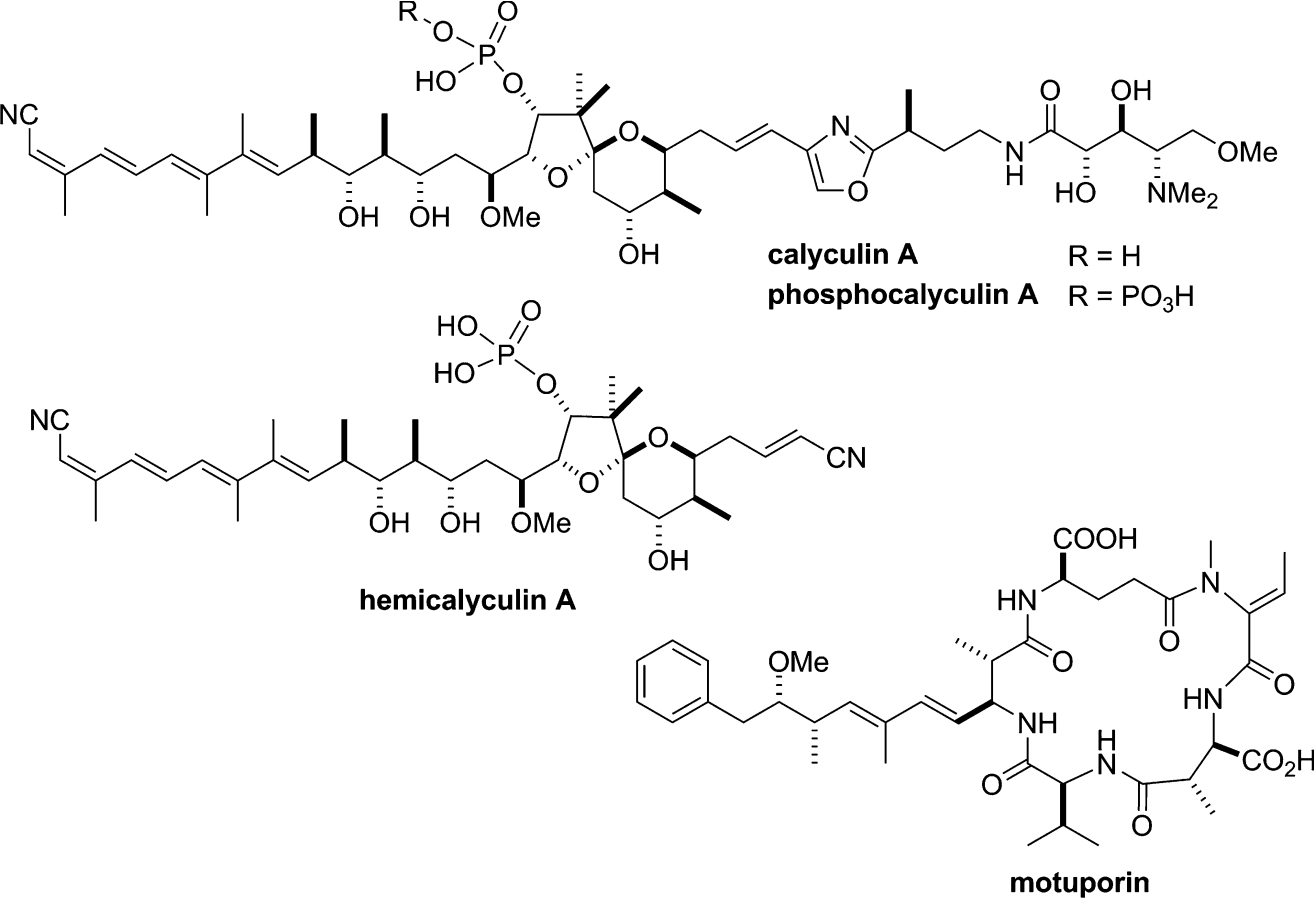
Chemical structures of calyculin A, phosphocalyculin A, hemicalyculin A and motuporin. For the structure of okadaic acid, see Fig. 4

Specific inhibitors against PP1 and 2A have been discovered from natural product origins, including cantharidin [127] from blister beetles, microcystins [85] from cyanobacteria, fostriecin [99] from actinomycetes, and okadaic acid [128] and calyculin [129] from marine sponges (Figs. 4, 5). This suggests that these protein phosphatases are the major targets for a diverse array of organisms in both marine and terrestrial environments, to confer a survival advantage by their inhibition, probably because these enzymes are common and essential for all eukaryotic cells. Notably, PP1/PP2A inhibitors are distributed not only in prokaryotes but also in eukaryotes, such as marine sponges. This raises the interesting question of why the latter organisms produce or accumulate phosphatase inhibitors inside their tissues, despite the coexistence of their own protein phosphatases. To address this intriguing question, the detailed biosynthetic mechanism is needed. Recent advances in next-generation sequencing have revealed the biosynthetic gene clusters of microcystin [130], fostriecin [131] and calyculin [132]. All of these inhibitors turned out to be biosynthesized by modular type enzymes, referred to as non-ribosomal peptide synthetases (NRPSs) and/or type I polyketide synthases (PKSs) [133]. This section briefly introduces the recent findings regarding the biosynthesis of natural phosphatase inhibitors derived from marine sponges.

Calyculin A (Fig. 5) was originally isolated as the major cytotoxic compound in the Japanese marine sponge *Discodermia calyx* [129]. Ishihara et al. [82] showed that, like okadaic acid, it specifically inhibited PP1 and 2A. The *K*_i_ values for the interaction of calyculin A with purified native PP1 and PP2A are reported to be 1 and 0.1 nM, respectively [81]. Structurally, calyculin A is composed of two different parts, with peptidic and polyketide backbones, and thus it was expected to be biosynthesized by an NRPS and PKS hybrid pathway. Interestingly, structure-activity relationship studies revealed that the peptidic backbone was not essential for the enzyme inhibition, but instead was required for potent cytotoxicity, probably because of the enhancement of membrane permeability [134]. This was confirmed by the crystal structure of the complex between a recombinant PP1 (γ isoform; PP1γ) and calyculin A, in which the peptidic portion was missing because of diverged electron density, suggesting the absence of interaction between the corresponding portion and PP1γ [135]. In contrast, the polyketide portion with the tetraene, 1,3-diol, and phosphate is essential and nearly sufficient for the phosphatase inhibition, as corroborated by the observation that hemicalyculin A (Fig. 5) inhibited PP1γ with *K*_i_ = 15 nM [135]. It is surprising that the truncated analog comprising only two-thirds of calyculin A retained such a high affinity.

In the case of calyculin biosynthesis, the biosynthetic gene cluster and the producing organism had remained unclear, in contrast to the PP inhibitors derived from microorganisms. This is because marine sponges harbor diverse symbionts, and thus there was speculation that the sponge-derived bioactive molecules are actually biosynthesized by symbiotic microorganisms. To obtain clues about the gene clusters in this complex consortium, the sponge metagenome is a suitable starting point. A PCR-based screening approach was performed on the sponge metagenomic DNA, with degenerate primers targeting the ketosynthase domain of the type I PKS. With a promising amplicon sequence in hand, the fosmid bearing the corresponding sequence was screened from the sponge metagenomic fosmid library. Finally, the PKS-NRPS hybrid gene cluster, ranging over 150 kb, was successfully obtained. The PKS-NRPS modular assembly line is in good agreement with the stepwise biosynthetic pathway of calyculin A [132].

Using the calyculin biosynthetic gene, the identification of the producer organism was performed. A single cell analysis employing laser microdissection in conjunction with a PCR analysis revealed that the ‘*Candidatus* Entotheonella’ sp., with a filamentous morphology, is the authentic producer of calyculin A [132]. The sponge symbiont ‘Entotheonella’ was originally identified from the sponge genus *Theonella*, which is closely related to *Discodermia*. Recently, ‘*Candidatus* Entotheonella factor’ was identified as the symbiotic producer of almost all bioactive molecules derived from *Theonella swinhoei*, collected in the waters surrounding Hachijo Island, Japan [136]. On the other hand, the Papua New Guinean *T. swinhoei* contains another phosphatase inhibitor, motuporin [88]. Even though its structure is a cyclic peptide related to the cyanobacterial microcystin, motuporin might also be one of Entotheonella’s metabolites. However, the biosynthetic gene cluster still remains unidentified. This new bacterium belonging to candidate phylum ‘Tectomicrobia’ is currently not culturable under normal laboratory conditions, and therefore a suitable host capable of heterologously expressing Entotheonella’s genes would be highly desirable [136].

To verify that the obtained biosynthetic gene cluster is actually responsible for calyculin biosynthesis, the functional analysis of the enzymes encoded in the calyculin biosynthetic gene cluster was conducted. There are three open reading frames annotated as phosphotransferases in the upstream region. Since a phosphate group is embedded in the structure of calyculin A, it was expected that at least one of the phosphotransferases could participate in the phosphorylation of dephosphonocalyculin A [137]. However, contrary to this hypothesis, an additional phosphorylation occurred in the enzyme reaction with calyculin A and CalQ, which is one of three phosphotransferases. The product had never been isolated from the marine sponge *D. calyx*, but it became apparent that the phosphorylation proceeded on the phosphate group to generate a pyrophosphate functionality by the structural elucidation of phosphocalyculin A (Fig. 5). As expected from the structure-activity relationship of calyculin A, the enzyme inhibition of phosphocalyculin A was significantly diminished. In addition, a dephosphorylation activity specific to phosphocalyculin A was detected in the cell-free extract of *D. calyx*, and it was triggered by tissue disruption. These results suggested that phosphocalyculin A is the true end product of the calyculin biosynthesis, but it had been overlooked because of enzymatic dephosphorylation during the extraction of the marine sponge. In the ecological context, the sponge symbiont ‘Entotheonella’ produces phosphocalyculin A as a protoxin, which is likely to be less toxic to the host sponge cells. Once the sponge tissue has been wounded, such as by predators, phosphatase and phosphocalyculin A are decompartmentalized to generate calyculin A in a quick and site-specific manner. This activated system meets both requirements: to avoid self-toxicity and to function as a chemical defense for a sessile animal [132, 138]. It is noteworthy that the activity of the phosphatase inhibitor calyculin A itself is regulated by enzymatic phosphorylation and dephosphorylation in the sponge-microbe association.

Extensive studies have been also conducted on okadaic acid (Fig. 4), which was originally isolated from the marine sponge *Halichondria okadai* [128]. Okadaic acid is one of the causative agents of diarrhetic shellfish poisoning (DSP) [139] and has also been detected in several different sponges, including the marine sponge *Suberites domuncula* and the freshwater sponge *Lubomirskia baicalensis* [140, 141]. It remains controversial whether okadaic acid is a symbiont-origin or dietary-derived metabolite, even though the dinoflagellates *Prorocentrum lima* and *P. hoffmannianum* have been identified as free-living producers of okadaic acid [142]. Okadaic acid is a highly potent inhibitor against PP1 and PP2A [78, 79, 81] (see “Effects of PP2A inhibitors on smooth muscle contraction” section, above), which are also common and essential enzymes for sponge cells. Indeed, okadaic acid-binding protein 1 (OABP1), exhibiting a phosphatase activity as well as a sequence similarity to PP2A, was isolated from *H. okadaii,* based on its affinity to okadaic acid [143]. The affinity-based fractionation concurrently afforded OABP2, with lower homology to protein phosphatase. Notably, the pretreatment of the sponge extract with cold acetone was required to detect the affinity of OABP2 to okadaic acid because of the tight binding to endogenous okadaic acid with a dissociation constant (*K*_d_) as low as 1 nM. Konoki et al. [144] successfully expressed recombinant OABP2, which allowed the confirmation of the nM order affinity to okadaic acid [144, 145]. Since OABP2 lacks not only phosphatase activity but also sequence similarity to any other proteins, its overall structure, capable of selectively accommodating okadaic acid, attracted keen interest. The X-ray structure of OABP2 in complex with okadaic acid was recently solved, at 1.4 Å resolution [146]. Remarkably, the overall conformation of OABP2 resembles that of aequorin [147], a Ca^2+^-binding photoprotein from *Aequorea aequorea*, despite the low sequence homology. Apoaequorin harbors coelenterazine to form active aequorin, which readily emits fluorescence in response to Ca^2+^. Moreover, OABP2 shares conformational similarity with other marine invertebrate proteins, such as the photoprotein berovin, the nonluminescent coelenterazine-binding protein (CBP) and the GTPase calexcitin, which all bind to small molecules. These findings are indicative of certain ecological functions of these proteins with a common overall fold.

## Conclusions

The two phosphorylation sites, Thr696 and Thr853 (human sequence numbering), of MYPT1 have drawn the most attention in the context of GPCR agonist-induced Ca^2+^ sensitization of smooth muscles. According to the current theory, these threonine residues are phosphorylated by RhoA/ROCK activated upon GPCR stimulation; phosphorylation of Thr696 is believed to inhibit MLCP activity to MLC, whereas that of Thr853 is thought to cause dissociation of MLCP from myosin and/or direct inhibition of MLCP activity. An increasing number of experimental observations appear to argue against this theory. However, it should be noted that our present knowledge and opinions totally rely upon the assumed specificity of antibodies. To gain a clearer view, the phosphorylation states of MYPT1 must be carefully re-evaluated using better means of quantification. Another critical factor regulating the MLCP activity is CPI-17, whose phosphorylation, expression and localization synergistically contribute to defining smooth muscle characteristics. CPI-17 may prove to be a very useful research tool as a specific MLCP inhibitor if it can be redesigned by biomolecular engineering for stabilizing the inhibitory potency induced by Thr38 phosphorylation.

The findings from the experiments with cultured smooth muscle cells and *staggerer* mutant mice consistently suggest that RORα plays an essential role in the vascular intrinsic clock mechanism that generates the circadian rhythm of vascular contractility. A scheme (Fig. 3) has been proposed where RORα regulates the circadian rhythm of the expression and activity of ROCK2, which then causes circadian oscillation of MYPT1 phosphorylation at T853 and MLC phosphorylation. Although the role of T853 phosphorylation in the regulation of MLCP activity is still controversial, the circadian oscillation of the MLCP activity is anyway likely to play a key role in the circadian oscillation of MLC phosphorylation. A similar vascular intrinsic clock may also underlie the mechanism for the endogenous circadian rhythm of blood pressure in humans.

Since the initial reports in 1989, the relaxant effect of okadaic acid on contractions of intact preparations has been shown in various smooth muscles. The recent findings obtained by the experiments using the highly specific PP2A inhibitors have led to the conclusion that, although there may be some tissue-specific factors in the responses of different smooth muscles, the relaxant effect is attributable to inhibition of PP2A. An important implication is that PP2A, as well as PP1, is somehow involved in the regulation and/or maintenance of the contractility of smooth muscles. Several pieces of evidence suggest that the site of action of PP2A is some rather downstream step of smooth muscle contraction such as cross-bridge cycling. Further studies are expected to elucidate the details of the function of PP2A in smooth muscles.

Although the overall picture of the biosynthetic mechanisms of sponge-derived protein phosphatase inhibitors is still largely unclear, recent comprehensive studies have illuminated some intriguing pieces of the complicated processes, such as producer microorganisms, binding proteins and dynamic bioconversions. It is particularly remarkable that the activity of the phosphatase inhibitor calyculin A is controlled by enzymatic phosphorylation and dephosphorylation of itself in the sponge-microbe association.
